# Seasonality Affects Fluid Intake Behaviors among Young Adults in Hebei, China

**DOI:** 10.3390/nu16111542

**Published:** 2024-05-21

**Authors:** Yongwei Lin, Na Zhang, Jianfen Zhang, Junbo Lu, Shufang Liu, Guansheng Ma

**Affiliations:** 1 Department of Nutrition and Food Hygiene, School of Public Health, Peking University, Beijing 100191, China; yweilin@bjmu.edu.cn (Y.L.); zjf@bjmu.edu.cn (J.Z.); ljb_breeze66@163.com (J.L.); 2 Laboratory of Toxicological Research and Risk Assessment for Food Safety, Beijing 100191, China; 3 National Institute for Nutrition and Health, Chinese Center for Disease Control and Prevention, Beijing 100050, China; 4 National Center for Occupational Safety and Health, Beijing 102308, China; 5 School of Public Health, Hebei University Health Science Center, Baoding 071000, China

**Keywords:** fluid intake, drinking fluid, water from food, seasonality, temperature

## Abstract

Background: Evidence on the association between environmental factors and fluid intake behavior remains limited. The current study aims to explore seasonal variations in fluid intake behaviors among young adults in China. Methods: A prospective cohort of 79 healthy young adults (43 males and 36 females) aged 19–21 in Hebei, China, was assessed for fluid intake behaviors for four seasons. For each assessment, the participants’ anthropometric measurements were collected. Temperature and humidity on survey days were measured. Participants’ total drinking fluid (TDF) was recorded using a self-administrative 7 d, 24 h fluid intake questionnaire. To calculate water from food (WFF), we weighed all foods consumed by participants. Duplicates of consumed food samples were collected to measure the water content via the drying method. Results: The mean total water intake (TWI) was 2761 ± 881, 2551 ± 845, 2210 ± 551, and 1989 ± 579 for spring, summer, fall, and winter, respectively (*F*(2.37) = 42.29, *p* < 0.001). The volume and proportion of TWI from TDF and WFF varied across the four seasons. The volume of WFF in spring (1361 ± 281, *F*(2.61) = 17.21, *p* < 0.001) and TDF in summer (1218 ± 502, *F*(2.62) = 9.36, *p* < 0.001) was among the highest, while participants’ fluid intake behaviors in spring and summer were less distinct than the other pairwise comparisons. A moderate association was found between outdoor temperature and TDF (*r* = 0.53, *p* < 0.01). Different general estimating equations suggested that gender, seasonality, outdoor temperature, differences in indoor and outdoor temperature, and mean temperature were independent factors of TDF. An interactive effect was found for gender and temperature, showing that the expected TDF of males may increase more as the temperature climbs. Conclusions: Gender, seasonality, and air temperature could significantly affect fluid intake behaviors, including the amount and type of fluid intake. However, the independent effect of BMI and humidity remains unclear.

## 1. Introduction

The survival and well-being of humans rely heavily on water, a precious natural resource and essential nutrient. Water is a fundamental building material of the human body, accounting for approximately 60% of an adult’s body mass, and widely distributed as body fluid in diverse cells, tissues, and organs [1]. In addition to regulating the body temperature and maintaining electrolyte balance, water is also deeply involved in human metabolism. Any disruption in the water homeostasis within the body, either dehydration or hyperhydration, may lead to adverse health effects [1].

Therefore, the brain closely monitors and regulates one’s plasma and serum osmolality in collaboration with the kidney and arginine vasopressin (AVP), a hormone released by the hypothalamus and posterior pituitary. When the body is dehydrated, either caused by excessive fluid loss or inadequate fluid intake, AVP is released to increase the water permeability of the distal tubule and collecting duct to promote water reabsorption. Meanwhile, thirst neurons in the anterior cingulate and insular cortex send out thirst signals that urge fluid intake behavior via a negative feedback loop [2]. If the hydration persists, one could have impeded cognitive function, attention, and working memory in the short term. Excessive water intake or a decrease in plasma or serum osmolality would inhibit AVP release, enabling the excretion of excessive fluid through urination. Hyperhydration, in rare cases, could result in water intoxication characterized by headache, nausea, impaired memory, and even death [3].

Current evidence suggests that dehydration is a significant threat to human health, yet it is frequently ignored or underestimated. In addition to the acute impacts of dehydration on one’s cognitive function as listed above, chronic dehydration can cause kidney problems and is associated with increasing incidences of a wide range of urological, gastrointestinal, circulatory, neurological, and metabolic disorders [3]. Adequate and regular fluid intake is the most practical approach to prevent dehydration. However, Sims et al. [4] reported that only 58% of US males, 48% of US females, 54% of Australian males, and 48% of Australian females met the recommended daily water intake, respectively. In an investigation conducted among young male athletes in China, only 56% and 37.6% of participants met the total drinking fluid (TDF) (1700 mL) and total water intake (TWI) (3000 mL) recommended by the Chinese Nutrition Society (CNS) [5]. Similar issues seem worldwide even though the recommendation for adequate fluid intake may vary across countries and organizations, therefore urging more effort to improve hydration status and public knowledge of proper hydration, especially among vulnerable populations.

Under the influence of physiological, environmental, and cultural factors, one’s fluid intake behavior may vary, as well as fluid excretion and hydration state, as a consequence. Temperature and humidity, in particular, may significantly impact physical activity level, an important factor in the fluid intake behavior and hydration state. A study of 573 healthy European adults found that higher temperatures were associated with lower physical activity levels, increased plasma and urine osmolality, and induced non-renal water loss, such as perspiration and respiratory loss [6]. As research evidence suggests, in addition to TWI and fluid loss, temperature and humidity may affect the distribution of different fluid sources. Tani et al. [7] found that the TWI was the highest in the summer (2331 mL/d) and lowest in the winter (2134 mL/d) among Japanese citizens. As the temperature climbed, the amount of water from food decreased, and drinking fluid increased.

In addition to the increasing amount of insensible water loss through exhalation and perspiration as the temperature climbs and the humidity drops, the physiological adaptation to environmental change might also partially contribute to variations in fluid intake behaviors across seasons. Several studies have discussed the underlying hormonal changes. Timpka et al. [8] found a J-shaped non-linear association between copeptin (CPP), a surrogate marker for circulating AVP, and the outdoor temperature that the log CPP z-score reached the nadir at 14.3 °C. Previously, they also discovered a distinct seasonal variation of CPP, with a peak in winter and a nadir in late summer [9]. Goswami et al. [10] found that concentrations of AVP and aquaporin-2 (AQP-2), a biomarker for the renal system response to AVP, were inversely correlated and significantly varied across seasons. They later concluded that the seasonal behavior of AVP and AQP-2 release is the premise of intracellular and extracellular fluid homeostasis [11].

Ocean currents, wind zones, air pressure bands, and varied topography shape the unique climate of different continents and regions in the vast expanse of the Earth. However, research and data on the effects of environmental factors, including temperature, humidity, and seasonality, remain limited. Current recommendations for water intake are established based on a mild climate and light physical activity. Little evidence has found application in establishing and refining recommended water intake worldwide. Therefore, we assessed the fluid intake behavior across all four seasons among young adults in Baoding, Hebei, China, hoping to provide supporting data for refining recommended water intake based on environmental factors and to evaluate its intensity and direction of influence on fluid intake behaviors.

## 2. Materials and Methods

### 2.1. Study Designs

A prospective cohort study on fluid intake behaviors throughout the four seasons was implemented in 2021 in Baoding, Hebei, China. Each assessment lasted seven consecutive days and followed the same procedure. The four assessments were conducted once each season from 12 to 27 April, 5 to 11 June, 21 to 27 October, and 9 to 15 December, respectively. The division of seasons follows the national standard (GB/T 42074-2022), in which the 5 d moving average of temperatures in spring, summer, fall, and winter are 10–22 °C, ≥22 °C, 10–22 °C, and <10 °C, respectively [12].

### 2.2. Ethics 

This study has been registered with the Chinese Clinical Trial Registry. The registration number is ChiCTR2100045268.

The Peking University Institutional Review Committee has reviewed and approved the study protocol. The ethical approval project identification code is IRB00001052-21013.

### 2.3. Participants

Healthy male and female college students aged 18–25 from a university in Baoding, Hebei, China, were recruited by convenient and snowball sampling. Participants who were smokers, habitually consumed alcohol (>20 g/d), and who had gastrointestinal, oral, and other chronic diseases were excluded from this study. All participants gave informed consent.

Required sample sizes were calculated using the following formula for a cross-sectional study: $N={\left(\frac{\mu_{\alpha /2}\sigma }{\delta }\right)}^{2}$, in which *α* = 0.05, *μ_α_*_/2_ = 1.96. According to previous studies on total drinking fluid among college students in Hebei, the total drinking fluid of the target population was 1135 ± 620 mL in spring and 342 ± 468 mL in winter [13,14]. Therefore, the estimated standard error (σ) of total fluid intake among college students in Hebei was 625, and *δ* was 158, with an estimated 20% drop-out rate. $N={\left(\frac{1.96\;\times \;625}{158}\right)}^{2}\times \left(1+20\%\right)=73.2$. The required sample size was 74.

### 2.4. Anthropometric Measurements

The height and weight of each participant were measured by trained investigators using a height–weight meter (HDM-300; Huaju, Zhejiang, China). Participants were required to wear light clothing and be barefoot. The height and weight were measured twice, and the mean was recorded to the nearest 0.1 cm and 0.1 kg, respectively. Each participant’s body mass index (BMI) was then calculated with the following equation: BMI = weight (kg)/height^2^ (m). The classification of normal (18.5 ≤ BMI < 24.0), underweight (<18.5), overweight (24.0 ≤ BMI < 28.0), and obese (≥28.0) was based on the criteria suggested by the Working Group on Obesity in China [15].

### 2.5. Temperature and Humidity

Temperature hygrometers (No.8813, Deli, Zhejiang, China) were placed on the sports ground, classroom, and dormitory. The indoor and outdoor measurements were recorded to the nearest 0.1 °C and 1% by trained investigators thrice daily at 10:00, 14:00, and 20:00.

### 2.6. Assessment of Fluid Intake

Total fluid intake is composed of drinking fluid and water from food. Drinking fluid intake was assessed for 7 d, and water from food was assessed for 3 d.

#### 2.6.1. Drinking Fluid

For each assessment, participants completed a self-administrative 7 d, 24 h fluid intake questionnaire previously used and tested for reliability and validity [16,17]. The questionnaire records the type, volume, time, and place of drinking fluid as described in a previous study [13]. TDF was classified into plain water and other beverages, including tea, sugar-sweetened beverages (SSBs), and alcohol. The classification of drinking fluid follows the General Standard for Beverages of China (GB/T 1-789-2015) [18]. The volume of drinking fluid was measured to the nearest 5 mL with a graduated water bottle. Each participant was asked to use a 5-mL-scaled bottle designed for the study when they drank any fluid to ensure accuracy.

#### 2.6.2. Water from Food 

Each assessment measured and recorded water from food (WFF) for three consecutive days. The exact amount of each food item consumed was weighed to assess WFF. All consumed foods were weighed before and after participants consumed them. Duplicates of consumed food items were collected. Moisture in collected food samples was measured via the drying method, according to the national standard of GB 5009.3-2016 [19]. WFF was then calculated using the amount of food consumed and the moisture content of each food item. Types of foods were classified as follows: staple food, dishes, porridge, soup, dairy, and snacks. The volume of dairy consumed (milk and yogurt) was recorded in the 7 d, 24 h fluid intake questionnaire. Moisture in dairy, fruits, and snacks was evaluated according to the China Food Composition Table (6th edition) [20]. 

### 2.7. Statistics

SPSS 26.0 was used for statistical analysis of baseline characteristics and fluid intake. All statistical tests were two-sided; a significance level of <0.05 represents a statistically significant difference. Continuous variables were analyzed using the *t*-test, one-way analysis of variance (ANOVA), and the non-parametric Kruskal–Wallis H-test. Results are presented as mean ± standard deviation ($\bar{x}$ ± S).

Repeated-measure analysis of variance (RMANOVA) was implemented to assess the impact of seasons on repeatedly measured variables that were normally or approximately normally distributed. For variables that did not follow a normal distribution, Friedman’s M test, the non-parametric RMANOVA, was implemented to assess the between-group differences by ranks. The Bonferroni-adjusted *p*-value was applied to post hoc comparisons.

To assess the association between environmental factors and total drinking fluid (TDF), we calculated the daily average of TDF, frequency, volume consumed each time, plain water intake, and beverage intake for the overall, male, and female participants based on data collected from the 4 × 7 d assessment on drinking fluid intake. Spearman rank correlations were then conducted between the collected 4 × 7 d of environmental measurements and the variables computed above. Scatter plots were also built. The general estimating equations (GEEs) with the working correlation matrix set to be the exchangeable mode were implemented to access the exact direction and the strength of the association between the environmental factors and TDF of participants while controlling for confounders.

## 3. Results

### 3.1. Characteristics of Participants

Table 1 summarizes the anthropometric measurements of participants in each season. Among 84 recruited participants aged 19 to 21, 94% (*n* = 79, 43 males and 36 females) completed all four assessments on fluid intake behavior from April to December 2021. For the 79 participants, the average age was 19.9 years old. Anthropometric measurements were taken each season. The mean height, weight, and BMI were 169.6 cm, 66.4 kg, and 23.0 kg/m^2^, respectively. In contrast to height and weight, BMI only exhibited significant gender differences in fall and winter, though the marginal *p*-value in spring and summer suggested a trend of statistical significance.

Results of MANOVA showed significant variation in participants’ height, weight, and BMI in different seasons. Pairwise comparisons suggested that the height in fall significantly differed from the rest of the seasons. No significant difference between fall and winter was found in terms of weight. BMI in each season was significantly different from the other season. Appendix A summarizes the significance levels for all pairwise comparisons among anthropometric measurements in different seasons.

### 3.2. Total Fluid Intake

Table 2 shows temperature and humidity during each survey period. Participant’s TFI, including TDF and WFF, is summarized in Table 3. We found significant impacts of seasonality, gender, and BMI on TFI, TDF, and WFF. We found that TFI, TDF, and WFF significantly differed between males and females every season. However, no significant impact of BMI on TDF in fall and WFF in spring was found, and the significance levels of pairwise comparisons for different BMIs are shown in Table A1. Seasonal variations of TWI (*F*(2.37) = 42.29, *p* < 0.001), TDF (*F*(2.62) = 9.36, *p* < 0.001), and WFF (*F*(2.61) = 17.21, *p* < 0.001) were found. The significance levels for all pairwise comparisons are summarized in Appendix A.

**Figure 1:**
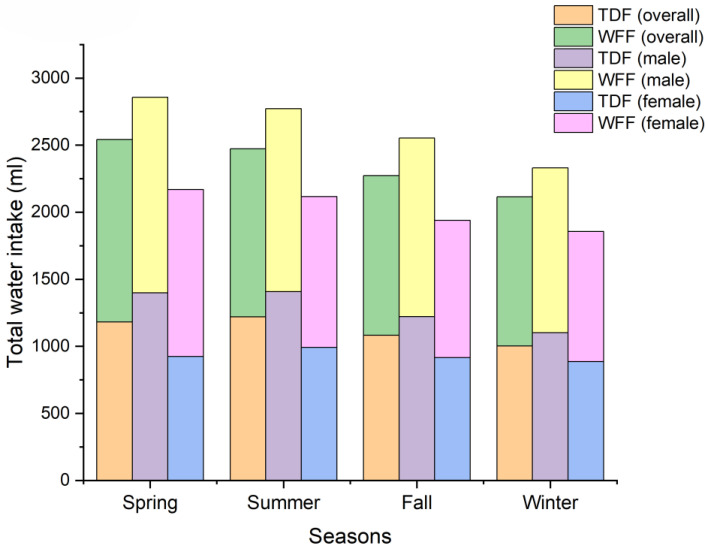
Participants’ total water intake among different seasons.

Seasonal variations in the composition of TWI were also analyzed. Figure 1 shows the TWI and its composition among different seasons. The volume of each fluid source consumed in different seasons is shown in Table 4, and the proportions are shown in Table 5. We found that the compositions of fluid sources significantly varied across the year. However, seasonal variations were found in the proportion but not the volume of water from staple foods. Neither the volume nor the proportion of dairy significantly varied across the year. We summarize the significance levels for all pairwise comparisons in Appendix A.

### 3.3. Environmental Factors and TDF

**Figure 2:**
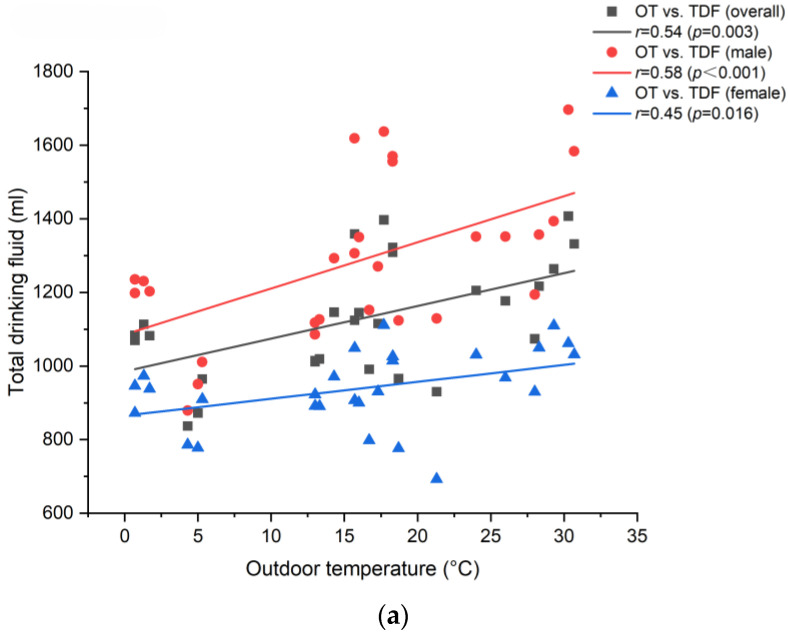

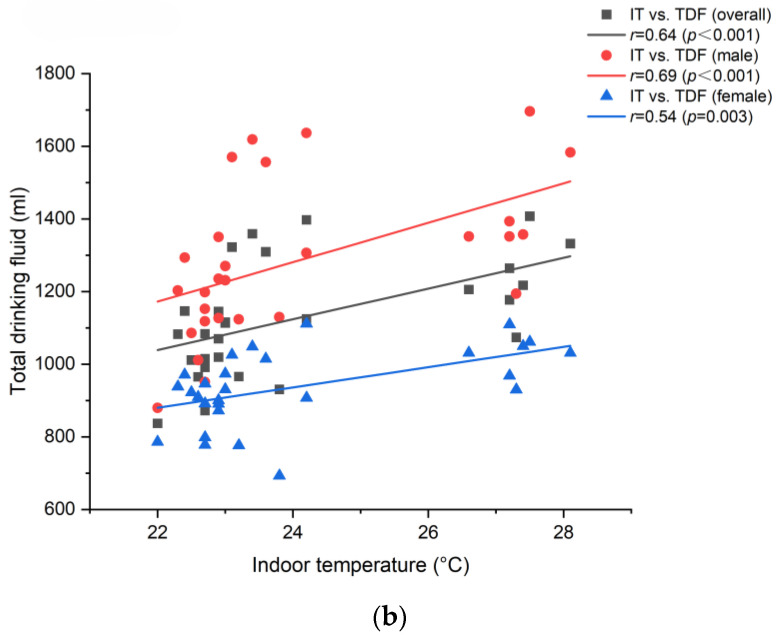
(**a**) Correlational plot between outdoor temperature and total fluid intake. (**b**) Correlational plot between indoor temperature and total fluid intake.

Correlational coefficients of drinking fluid and environmental factors derived from Spearman rank correlation are listed in Table 6. We found that outdoor and indoor temperatures were significantly associated with TDF, volume consumed each time, and plain water intake. MT and ΔT were also associated with TDF. No significant correlation between temperatures and the frequency of fluid intake was found for the overall population, but moderate correlations were found when analyzing the average frequency of male and female participants separately. However, beverage intake had a stronger association with indoor temperature than outdoor temperature. Most of the correlational coefficients exhibited gender differences. Participants’ TDF, frequency of fluid intake, volume consumed each time, and plain water intake tended to increase as indoor and outdoor temperatures climbed. Scatter plots of temperature and total drinking fluid, as shown in Figure 2, were also built to visualize the correlation between environmental factors and the fluid intake behavior of participants.

GEEs were built based on 4 × 7 d data for seasons, outdoor temperature, ΔT, and MT, with TDF as the dependent variable, as shown in Table 7. The models were built separately to avoid collinearity. We found that gender possibly confounded the association between BMI and TDF among the study population. Moreover, the temperature was the potential confounder of the association between humidity and TDF (β = 4.6, *p* = 0.002). ΔT (β = 4.8, *p* = 0.008), and MT (β = 15.4, *p* < 0.001) were all positively associated with TDF. TDF increased as the temperature climbed. The results of the GEE analysis also suggested that the interactions between gender and outdoor temperature, as well as the differences in temperature, and that the association between temperature and TDF might be stronger among males than females. Seasons were also associated with TDF, among which summer (β = 236.1, *p* < 0.001) has the leading β, followed by spring (β = 178.7, *p* < 0.001) and summer (β = 79.0, *p* = 0.018), respectively.

## 4. Discussion

In the current study, we assessed the fluid intake behaviors of the same group of participants four times throughout 2021, one in each season. Recruited participants were young adults aged 19–21 at a university in Hebei, China.

In each assessment, participants’ anthropometric measurements were also obtained. When analyzing participants’ anthropometric measurements, we found significant seasonal variations in participants’ weight, height, and BMI as consequences. The variations in participants’ height and weight could also be due to minor deviations in the timing and instrumental reading while measuring in different seasons. Significant variations in fluid intake behaviors of participants with different weight statuses were found when conducting one-way ANOVA, and obese participants tended to have higher TDF and TWI than the rest. However, the association between BMI and TDF could be potentially confounded by gender due to the significant differences in the BMI of male and female participants and the strong collinearity of gender and BMI while we conducted the GEE analysis. 

Participants’ fluid intake behaviors, including the TDF, WFF, and TWI, exhibited significant gender differences in all four seasons. Male participants in our study tended to have higher TDF and WFF than females, resulting in a higher TWI. In line with the results from the present study, a recent survey among 2233 Chinese residents in 27 cities reported a significantly higher TFI in males than females regardless of age group [21]. Wu et al. [22] also found a significant difference in daily TWI between US males and females based on the National Health and Nutrition Examination Survey 2011–2014 data. According to the results of the GEE analysis, gender was significantly associated with TDF. Males exhibited higher coefficients than females, ranging from 221.0 ± 87.8 to 428.2 ± 105.0 depending on the other variable in the model built.

The higher muscle contents in the body composition of males might contribute to the gender differences in fluid intake behaviors and requirements. Established guidelines of fluid intake worldwide also embody the gender difference. For instance, the recommended TWI by the CNS is 3000 mL/d for males and 2700 mL/d for females. The recommended TDF by the CNS were 1700 mL/d and 1400 mL/d for males and females [23]. European Food Safety Authority (EFSA) recommended a TWI of 2500 mL/d for males and 2000 mL/d for females [24]. However, neither the mean TDF of males nor females across the four seasons in the current study met the recommendation of CNS and EFSA. The mean TWI for both male and female participants was also lower than that recommended by CNS throughout the year, urging attention to the importance of adequate fluid intake and establishing an optimal fluid intake habit among the general population.

Regarding environmental factors, we measured and recorded indoor and outdoor temperature and humidity of all 28 survey days. The mean and differences between indoor and outdoor temperature and humidity were also calculated. The statistical analysis suggested that all measurements of temperature significantly varied across different seasons but not that of humidity. Only the difference in indoor and outdoor humidity in summer significantly differed from the rest of the seasons. That could partially explain the negligible influence of humidity on TDF in our study.

According to the results of correlational analysis among daily temperature, humidity, and TDF, the temperature significantly impacted participants’ fluid intake behaviors, including the volume, frequency, and type of fluid consumed. Outdoor temperature, indoor temperature, and MT were all positively correlated with TDF. The GEE analysis indicated that TDF would increase as outdoor temperature, ΔT, or MT climbed. However, only indoor humidity was found to have a weak association with the volume consumed each time among all participants and the plain water intake among females. ΔH was also weakly correlated with the volume consumed each time and plain water intake among males.

Similarly, a previous study in Japan found that the temperature but not humidity were associated with water intake, that for every 1 °C increase in the mean air temperature, the expected TDF would increase by 8.4 g/d when controlled for BMI and gender [7]. The correlational analysis suggested a correlation between the temperature and humidity. For instance, indoor humidity correlated with indoor temperature (r = 0.375, *p* = 0.049) and ΔT (r = −0.460, *p* = 0.014). Even though the correlations were weak, summing up with the strong collinearity we discovered later in the GEE analysis, temperature might still confound the correlation between indoor humidity and TDF.

Fluid intake behaviors may also vary according to seasons, especially the volume and the composition of TWI. The results of MANOVA showed significant differences in TDF (*F*(2.62) = 9.36, *p* < 0.001), WFF (*F*(2.61) = 17.21, *p* < 0.001), and TWI (*F*(2.37) = 42.29, *p* < 0.001) across the four seasons. TDF in summer (1219 ± 502) was significantly higher than in fall (1082 ± 475, *p* = 0.015) and winter (1082 ± 475, *p* = 0.009) but did not significantly differ from that in spring (1182 ± 594, *p* = 1.000). As results from GEE analysis showed, when controlled for gender, summer (β = 236.1, *p* < 0.001) had the leading β among all seasons with winter as the reference. The β of spring and fall were 178.7 (*p* < 0.001) and 79.0 (*p* = 0.018), respectively. Consistent with our findings, Malisova et al. [25] concluded that there was no significant difference in the water balance between summer and winter among Greek residents, while TDF was significantly higher in summer than in winter, and the proportion of WFF in winter outran that in summer.

We also analyzed the volume and proportion of specific sources of fluid intake. The results indicated that the proportion of TWI from TDF was significantly higher in summer than in fall and winter. The volume but not the proportion of WFF in spring, specifically the volume of water from dishes and porridge, was significantly higher than in the resting seasons. However, Tani et al. [7] concluded that for every 1 °C increase in the mean air temperature, the expected WFF would decrease by 3.1 g/d. The contradicting results might be attributed to the significant increase in other beverage intake in summer. Correspondingly, a survey conducted in Korea showed that plain water and beverage intake increases by 19.7% and 7.6% in summer compared to spring, though we did not find a significant difference in the volume of plain water intake between spring and summer [26]. Possibly due to differences in dietary habits and culture, the proportion of WFF in TWI in the present study was also higher across the four seasons in our study than the data from previous assessments worldwide. For instance, only 18% of the TWI intake of US citizens were WFF based on 1999–2006 NHANES data [27]. The proportion of WFF in TWI among UK and French citizens was 27% and 36%, respectively [28].

Previous studies exploring the impact of seasonality, temperature, and humidity on fluid intake behaviors rarely conducted assessments in all four seasons. Otherwise, data from national-based dietary surveys were utilized and analyzed. Limitations and imprecisions could be found in measuring and estimating the exact amount of fluid intake. In the present study, composed of four assessments of the same group of participants, we employed a specifically designed 7 d, 24 h fluid intake questionnaire that had been proven reliable and valid in our past study. Participants were also trained to record the type, volume, time, and place of drinking fluid immediately after. The exact amount of WFF was calculated by weighting the consumed amount of each food item and measuring moisture in the duplicate of each food item via the drying method, significantly improving the accuracy and precision of TWI measurement. In addition, the current study analyzed the seasonal differences of TWI, TDF, and WFF and further investigated the association between participants’ fluid intake behaviors and environmental factors, including indoor and outdoor temperature and humidity. Limitations of the present study are that participants were prescribed to only participate in light physical activity for quality control. However, physical activity is essential in fluid intake behaviors and might be significantly influenced by the environment. By minimizing the confounding effect of physical activity, we also lose the chance to explore the impact of physical activity on participants’ fluid intake behaviors and the underlying interaction of environmental factors and physical activities, as little evidence has been established on the interactive effects of physical activity and environmental factors on one’s fluid intake behavior and overall hydration state.

## 5. Conclusions

The current study verified the association between fluid intake behaviors and environmental factors. Even though it was possibly due to the relatively steady humidity in our survey site across the year, no significant association was found between humidity and fluid intake behaviors, and seasonality and temperature could still significantly impact the total volume of fluid intake and the proportion of different fluid sources in TWI. Gender was the other factor significantly associated with fluid intake behaviors. Even though previous research suggested that overweight and obesity were associated with higher TWI, urine osmolality, and risk of dehydration, gender possibly confounded the correlation between BMI and fluid intake behaviors in the current study.

In subsequent studies, an in-depth exploration of one’s anthropometric measurements, including height, weight, BMI, body surface area, and blood pressure, should be completed, along with analyses of their association and water homeostasis within the body. TDF increases as temperature elevates and is the highest in summer. Males tended to have higher TWI, WFF, and TDF across all four seasons. Their TDF could also be impacted by temperature to a greater extent than that of females.

Future studies could incorporate biomarkers of hydration status into the analysis to obtain a more comprehensive and systematic understanding of how environmental factors may affect one’s hydration status and water homeostasis, thereby finding implications in refining the recommendation of fluid intake in different seasons.

## Figures and Tables

**Table 1:** Seasonal variations in characteristics of subjects.

		Spring	Summer	Fall	Winter	*df* (Adjusted)	*F*	*p*
Height (cm)	Overall	169.4 ± 8.2	169.4 ± 8.4	170 ± 7.9	169.5 ± 7.7	2.27	8.00	<0.001
	Male	174.7 ± 5.6	175.1 ± 5.6	174.8 ± 5.7	174.2 ± 5.5			
	Female	163.0 ± 5.9	162.7 ± 5.9	164.2 ± 6.2	163.9 ± 6.1			
	*t*	9.03	9.53	7.89	7.93			
	*p*	<0.001	<0.001	<0.001	<0.001			
Weight (kg)	Overall	65.8 ± 15.7	65.1 ± 15.3	67.2 ± 16.0	67.5 ± 16.1	1.85	40.83	<0.001
	Male	72.4 ± 15.2	71.7 ± 14.4	72.3 ± 14.8	74.5 ± 14.7			
	Female	57.9 ± 12.4	57.3 ± 12.5	58.8 ± 13.1	59.1 ± 13.5			
	*t*	4.58	4.71	4.84	4.82			
	*p*	<0.001	<0.001	<0.001	<0.001			
BMI (kg/m^2^)	Overall	22.8 ± 4.5	22.6 ± 4.4	23.1 ± 4.7	23.4 ± 4.8	2.06	29.96	<0.001
	Male	23.6 ± 4.5	23.3 ± 4.3	24.3 ± 4.5	24.5 ± 4.4			
	Female	21.8 ± 4.4	21.6 ± 4.4	21.8 ± 4.6	22.0 ± 4.8			
	*t*	1.85	1.76	2.38	2.40			
	*p*	0.068	0.083	0.020	0.019			

Statistics were derived from MANOVA. BMI, body mass index; *df*, degree of freedom.

**Table 2:** Air temperature and humidity in each season.

		Spring	Summer	Fall	Winter	*H*	*p*
Temperature (°C)	indoor	23.4 ± 0.5	27.3 ± 0.4	22.9 ± 0.6	22.6 ± 0.3	19.96	<0.001
	outdoor	18.1 ± 1.8	28.1 ± 2.4	14.7 ± 1.7	2.7 ± 2.1	24.70	<0.001
	mean	20.8 ± 1	27.7 ± 1.4	17.8 ± 1.0	12.7 ± 1.0	24.61	<0.001
	Δ	5.3 ± 1.7	1.8 ± 0.9	1.8 ± 1.5	19.9 ± 2.2	1.56	<0.001
Humidity (%)	indoor	47.4 ± 4.7	47.3 ± 10.0	44.0 ± 6.0	40.4 ± 3.7	5.78	0.123
	outdoor	55.3 ± 21.7	46.9 ± 21.7	52 ± 9.8	63.1 ± 16.4	2.77	0.428
	mean	51.4 ± 13.0	47.1 ± 15.8	48.0 ± 6.1	51.8 ± 9.7	23.97	0.669
	Δ	16.6 ± 7.1	9.6 ± 5.7	11.1 ± 6.7	23.0 ± 13.4	7.41	0.060

Statistics were derived from the Kruskal–Wallis H-test. Δ = outdoor − indoor, mean = (outdoor + indoor)/2.

**Table 3:** Seasonal variations in total fluid intake.

			Spring	Summer	Fall	Winter	*df* (Adjusted)	*F*	*p* ^a^
TDF (7 d)			1182 ± 594	1219 ± 502	1082 ± 475	1003 ± 394	2.62	9.36	<0.001
	Gender	Male (*n* = 43)	1398 ± 612	1408 ± 525	1221 ± 494	1101 ± 402			
		Female (*n* = 36)	924 ± 458	992 ± 365	916 ± 398	886 ± 356			
	*t*		3.83	4.01	2.98	2.49			
	*p* ^c^		<0.001	<0.001	0.004	0.015			
	BMI	Underweight (*n* = 10)	806 ± 423	914 ± 355	914 ± 417	777 ± 315			
		Normal (*n* = 41)	1110 ± 510	1088 ± 335	1014 ± 478	930 ± 349			
		Overweight (*n* = 15)	1569 ± 796	1533 ± 664	1276 ± 360	1193 ± 321			
		Obese (*n* = 13)	1252 ± 474	1502.6 ± 556	1205 ± 567	1191 ± 513			
	*F*		4.17	6.73	1.89	4.17			
	*p* ^b^		0.009	<0.001	0.138	0.009			
WFF (3 d)			1361 ± 281	1255 ± 353	1190 ± 289	1112 ± 383	2.61	17.21	<0.001
	Gender	Male	1458 ± 267	1364 ± 400	1331 ± 273	1230 ± 418			
		Female	1245 ± 256	1124 ± 229	1023 ± 209	970 ± 282			
	*t*		3.61	3.33	5.54	3.18			
	*p* ^c^		<0.001	0.001	<0.001	0.002			
	BMI	Underweight	1208.6 ± 205.0	1007 ± 143	946 ± 166	830 ± 171			
		Normal	1386.6 ± 302.4	1295 ± 315	1190 ± 268	1134 ± 360			
		Obese	1463.4 ± 293.2	1295 ± 514	1304 ± 297	1158 ± 441			
		Overweight	1280.9 ± 190.0	1274 ± 308	1247 ± 335	1204 ± 438			
	*F*		2.20	1.96	3.64	2.28			
	*p* ^b^		0.095	0.127	0.016	0.086			
TWI (3 d)			2716 ± 881	2551 ± 845	2210 ± 551	1989 ± 579	2.37	42.29	<0.001
	Gender	Male	3062 ± 936	2846 ± 951	2449 ± 534	2177 ± 602			
		Female	2304 ± 96	2198 ± 518	1923 ± 423	1764 ± 466			
	*t*		4.2	3.66	4.78	3.44			
	*p* ^c^		<0.001	<0.001	<0.001	<0.001			
	BMI	Underweight	2130 ± 579	1930 ± 499	1832 ± 431	1542 ± 318			
		Normal	2642 ± 634	2431 ± 506	2154 ± 526	1965 ± 548			
		Obese	3334 ± 1403	3008 ± 1430	2476 ± 558	2125 ± 465			
		Overweight	2688 ± 611	2879 ± 681	2368 ± 550	2250 ± 766			
	*F*		4.59	4.81	3.548	3.47			
	*p* ^b^		0.005	0.004	0.018	0.020			

Statistics were derived from RMANOVA ^a^, one-way ANOVA ^b^, and *t*-test ^c^. TDF: total drinking fluid; WFF: water from food; TWI: total water intake; BMI: body mass index; *df*: degree of freedom.

**Table 4:** Sources of fluid intake in different seasons (volume).

		Spring	Summer	Fall	Winter	*df* (Adjusted)	*F*	*p*
TDF (mL)		1182 ± 594	1219 ± 502	1082 ± 475	1003 ± 394	2.62	9.36	<0.001
	Plain water	1077 ± 579	1039 ± 489	979 ± 496	890 ± 413	2.66	6.72	<0.001
	Other beverages	106 ± 125	180 ± 170	103 ± 127	113 ± 159	2.64	8.60	<0.001
WFF (mL)		1361 ± 281	1255 ± 353	1190 ± 289	1112 ± 383	2.61	17.21	<0.001
	Staple foods	257 ± 96	234 ± 122	244 ± 104	214 ± 111	2.78	7.46	<0.001
	Dishes	542 ± 132	493 ± 169	484 ± 120	484 ± 156	2.91	7.09	<0.001
	Soup	117 ± 103	143 ± 137	134 ± 107	92 ± 134	2.94	4.27	0.006
	Porridge	197 ± 119	153 ± 131	138 ± 104	147 ± 174	2.58	5.69	0.002
	Dairy	32 ± 61	26 ± 60	17 ± 36	19 ± 58	2.62	1.61	0.195
	Snacks	216 ± 68	206 ± 81	173 ± 73	155 ± 85	2.85	16.52	<0.001

Statistics were derived from RMANOVA. TDF, total drinking fluid; WFF, water from food; *df*, degree of freedom.

**Table 5:** Sources of fluid intake in different seasons (proportions).

		Spring	Summer	Fall	Winter	*M*	*p*
TDF (%)		47.4 ± 12.2	49.3 ± 11.9	44.9 ± 11.8	43.8 ± 11.8	12.54	0.006
	Plain water	91.5 ± 11.8	84.1 ± 18.3	87.4 ± 17.0	85.7 ± 20.4	13.42	0.004
	Other beverages	8.6 ± 11.8	15.9 ± 18.3	12.7 ± 17.0	14.3 ± 20.4	13.42	0.004
WFF (%)		52.6 ± 12.2	51.2 ± 14.2	55.1 ± 11.8	56.2 ± 11.8	8.42	0.038
	Staple foods	18.7 ± 5.4	18.3 ± 6.8	20.2 ± 5.9	19.4 ± 7.5	6.90	0.075
	Dishes	39.9 ± 5.7	39.3 ± 7.5	41.1 ± 6.3	44.8 ± 9.2	21.35	<0.001
	Soup	8.4 ± 7.2	11.0 ± 9.9	10.9 ± 7.9	7.8 ± 10.7	16.05	0.001
	Porridge	14.9 ± 9.2	12.7 ± 11.4	11.9 ± 9.3	12.5 ± 13.8	8.29	0.040
	Dairy	2.3 ± 3.9	2.0 ± 4.5	1.5 ± 3.2	1.5 ± 4.2	4.69	0.196
	Snacks	15.8 ± 3.6	16.8 ± 6.9	14.3 ± 5.1	13.9 ± 7.5	21.41	0.001

Statistics were derived from Friedman’s M test. TDF, total drinking fluid; WFF, water from food.

**Table 6:** Correlation among daily temperature, humidity, and total drinking fluid.

		Temperature				Humidity			
		Outdoor	Indoor	Mean	|Δ|	Outdoor	Indoor	Mean	|Δ|
TDF	Overall	0.54 **	0.64 **	0.56 **	−0.43 *	−0.18	0.21	−0.13	−0.26
	Male	0.58 **	0.69 **	0.60 **	−0.48 **	−0.19	0.23	−0.14	−0.21
	Female	0.45 *	0.54 **	0.48 *	−0.37 **	−0.18	0.12	−0.13	−0.24
Frequency	Overall	0.06	0.34	0.10	−0.01	−0.25	−0.12	−0.26	−0.04
	Male	0.47 *	0.66 **	0.51 **	−0.44 *	−0.16	0.23	−0.09	−0.22
	Female	0.52 **	0.56 **	0.55 **	−0.46 *	−0.14	0.26	−0.10	−0.10
Volume consumed each time	Overall	0.75 **	0.62 **	0.75 **	−0.68 **	−0.02	0.40 *	0.05	−0.29
	Male	0.74 **	0.73 **	0.74 **	−0.71 **	−0.14	0.32	0.06	−0.48 **
	Female	0.80 **	0.67 **	0.79 **	−0.72 **	−0.11	0.35	−0.05	−0.28
Plain water	Overall	0.44 *	0.52 **	0.46 *	−0.34	−0.17	0.23	−0.13	−0.21
	Male	0.62 **	0.72 **	0.64 **	−0.56 **	−0.17	0.34	−0.09	−0.39 *
	Female	0.69 **	0.62 **	0.70 **	−0.61 **	−0.08	0.42 *	−0.02	−0.19
Other beverages	Overall	0.43 *	0.48 **	0.44	−0.33	−0.13	−0.07	−0.10	−0.20
	Male	0.47 *	0.51 **	0.48 **	−0.45 *	−0.07	0.00	−0.06	−0.17
	Female	0.29	0.38 *	0.32	−0.21	−0.20	−0.10	−0.17	−0.28

Statistics were derived from Spearman rank correlation. TDF, total drinking fluid Δ = outdoor − indoor. * The significance level is <0.05; ** the significance level is <0.01.

**Table 7:** Variables associated with total drinking fluid.

			β	SE	95% CI	*p*
Model 1	Intercept		814.7	62.6	(692.1–937.3)	<0.001
	Gender	Female	Reference			
		Male	346.6	89.5	(171.1–522.0)	<0.001
	Seasons	Winter	Reference			
		Spring	178.7	53.9	(73.0–284.4)	<0.001
		Summer	236.1	44.6	(148.7–323.5)	<0.001
		Fall	79.0	33.5	(13.3–144.7)	0.018
Model 2	Intercept		865.2	59.9	(747.8–982.7)	<0.001
	Gender	Female	Reference			
		Male	221.0	87.8	(48.8–393.1)	0.012
	OT		4.6	1.5	(1.6–7.6)	0.002
	OT × Gender	OT × Female	Reference			
		OT × Male	7.9	3.3	(1.4–14.4)	0.017
Model 3	Intercept		977.4	64.7	(850.5–1104.3)	<0.001
	Gender	Female	Reference			
		Male	428.2	105.0	(222.3–634.0)	<0.001
	ΔT		4.8	1.8	(1.3–8.4)	0.008
	ΔT × Gender	ΔT× Female	Reference			
		ΔT× Male	9.9	4.0	(2.1–17.6)	0.012
Model 4	Intercept		630.8	82.1	(469.8–791.8)	<0.001
	Gender	Female	Reference			
		Male	345.9	89.5	(170.5–521.4)	<0.001
	MT		15.4	3.0	(9.4–21.4)	<0.001

OT, outdoor temperature; ΔT = outdoor temperature − indoor temperature; MT = (outdoor temperature + indoor temperature)/2; SE, standard error; CI, confidence interval.

## Data Availability

Data described in the manuscript, code book, and analytic code will be available upon reasonable request due to privacy.

## Abbreviations

Analysis of variance	(ANOVA)
Aquaporin-2	(AQP-2)
Arginine vasopressin	(AVP)
Body mass index	(BMI)
Chinese Nutrition Society	(CNS)
Copeptin	(CPP)
Differences in indoor and outdoor temperature	(ΔT)
European Food Safety Authority	(EFSA)
General estimating equations	(GEE)
Mean of indoor and outdoor temperature	(MT)
Repeated-measure analysis of variance	(RMANOVA)
Sugar-sweetened beverages	(SSBs)
Total water intake	(TWI)
Total drinking fluid	(TDF)
Water from food	(WFF)

## Appendix A

**Table A1:** Significance levels of pairwise comparisons among participants with different BMI.

		TWI				WFF				TDF			
		Underweight	Normal	Overweight	Obese	Underweight	Normal	Overweight	Obese	Underweight	Normal	Overweight	Obese
Spring	Underweight		0.262	0.002	0.411		0.423	0.157	1.000		0.767	0.008	0.375
	Normal	0.262		0.057	1.000	0.423		1.000	1.000	0.767		0.050	1.000
	Overweight	0.002	0.057		1.000	0.157	1.000		0.503	0.008	0.050		0.840
	Obese	0.411	1.000	1.000		1.000	1.000	0.503		0.375	1.000	0.840	
Summer	Underweight		0.259	0.005	0.012		0.128	0.275	0.428		1.000	0.008	0.017
	Normal	0.259		0.137	0.330	0.128		1.000	1.000	1.000		0.010	0.032
	Overweight	0.005	0.137		1.000	0.275	1.000		1.000	0.008	0.010		1.000
	Obese	0.012	0.330	1.000		0.428	1.000	1.000		0.017	0.032	1.000	
Fall	Underweight		0.530	0.015	0.090		0.085	0.013	0.069		1.000	0.366	0.854
	Normal	0.530		0.183	1.000	0.085		1.000	1.000	1.000		0.398	1.000
	Overweight	0.015	0.183		1.000	0.013	1.000		1.000	0.366	0.398		1.000
	Obese	0.090	1.000	1.000		0.069	1.000	1.000		0.854	1.000	1.000	
Winter	Underweight		0.168	0.014	0.010		0.145	0.209	0.120		1.000	0.047	0.060
	Normal	0.168		0.611	0.447	0.145		1.000	1.000	1.000		0.130	0.182
	Overweight	0.014	0.611		1.000	0.209	1.000		1.000	0.047	0.130		1.000
	Obese	0.010	0.447	1.000		0.120	1.000	1.000		0.060	0.182	1.000	

TWI, total water intake; WFF, water from food; TDF, total drinking fluid. One-way ANOVA with Bonferroni adjustment for multiple comparisons.

**Table A2:** Significance levels of pairwise comparisons among different seasons.

		Spring	Summer	Fall	Winter
TDF	Spring		1.000	0.220	0.009
	Summer	1.000		0.015	<0.001
	Fall	0.220	0.015		0.130
	Winter	0.009	<0.001	0.130	
WFF	Spring		0.002	<0.001	<0.001
	Summer	0.002		0.084	<0.001
	Fall	<0.001	0.084		0.064
	Winter	<0.001	<0.001	0.064	
TWI	Spring		1.000	<0.001	<0.001
	Summer	1.000		0.010	<0.001
	Fall	<0.001	0.010		0.024
	Winter	<0.001	<0.001	0.024	

TDF, total drinking fluid; WFF, water from food; TWI, total water intake. RMNOVA with Bonferroni adjustment for multiple comparisons.

**Table A3:** Significance levels of pairwise comparison among different sources of fluid intake (volume).

		Spring	Summer	Fall	Winter			Spring	Summer	Fall	Winter
TDF	Spring		1.000	0.220	0.009	WFF	Spring		0.014	<0.001	<0.001
	Summer	1.000		0.015	<0.001		Summer	0.014		0.505	<0.001
	Fall	0.220	0.015		0.130		Fall	<0.001	0.505		0.384
	Winter	0.009	<0.001	0.130			Winter	<0.001	<0.001	0.384	
Plain water	Spring		1.000	0.267	0.004	Staple foods	Spring		0.162	0.886	<0.001
	Summer	1.000		1.000	0.003		Summer	0.162		1.000	0.093
	Fall	0.267	1.000		0.088		Fall	0.886	1.000		0.009
	Winter	0.004	0.003	0.088			Winter	<0.001	0.093	0.009	
Other beverages	Spring		<0.001	1.000	1.000	Dishes	Spring		0.010	<0.001	0.002
	Summer	<0.001		<0.001	0.012		Summer	0.010		1.000	1.000
	Fall	1.000	<0.001		1.000		Fall	<0.001	1.000		1.000
	Winter	1.000	0.012	1.000			Winter	0.002	1.000	1.000	
						Soup	Spring		0.537	1.000	0.682
							Summer	0.537		1.000	0.026
							Fall	1.000	1.000		0.064
							Winter	0.682	0.026	0.064	
						Porridge	Spring		0.030	<0.001	0.038
							Summer	0.030		1.000	1.000
							Fall	<0.001	1.000		1.000
							Winter	0.038	1.000	1.000	
						Snacks	Spring		1.000	<0.001	<0.001
							Summer	1.000		0.002	<0.001
							Fall	<0.001	0.002		0.685
							Winter	<0.001	<0.001	0.685	

TDF, total drinking fluid; WFF, water from food. RMANOVA with Bonferroni adjustment for multiple comparisons.

**Table A4:** Significance levels of pairwise comparison among different sources of fluid intake (proportion).

		Spring	Summer	Fall	Winter			Spring	Summer	Fall	Winter
%TDF	Spring		1.000	0.292	0.387	%WFF	Spring		1.000	0.337	0.444
	Summer	1.000		0.019	0.028		Summer	1.000		0.136	0.186
	Fall	0.292	0.019		1.000		Fall	0.337	0.136		1.000
	Winter	0.387	0.028	1.000			Winter	0.444	0.186	1.000	
%Plain water	Spring		0.010	0.201	0.787	%Soup	Spring		1.000	0.313	0.577
	Summer	0.010		1.000	0.615		Summer	1.000		1.000	0.053
	Fall	0.201	1.000		1.000		Fall	0.313	1.000		0.002
	Winter	0.787	0.615	1.000			Winter	0.577	0.053	0.002	
						%Dishes	Spring		1.000	1.000	0.005
							Summer	1.000		0.292	<0.001
							Fall	1.000	0.292		0.082
							Winter	0.005	<0.001	0.082	
						%Porridge	Spring		0.147	0.201	0.097
							Summer	0.147		1.000	1.000
							Fall	0.201	1.000		1.000
							Winter	0.097	1.000	1.000	
						%Snacks	Spring		1.000	0.444	0.010
							Summer	1.000		0.023	<0.001
							Fall	0.444	0.023		1.000
							Winter	0.010	<0.001	1.000	

TDF, total drinking fluid; WFF, water from food Friedman’s M test with Bonferroni adjustment for multiple comparisons.
